# Interleukin-35 expression is associated with colon cancer progression

**DOI:** 10.18632/oncotarget.17751

**Published:** 2017-05-10

**Authors:** Jian Zhang, Tao Mao, Shuyun Wang, Dongsheng Wang, Zhaojian Niu, Zhenqing Sun, Jianli Zhang

**Affiliations:** ^1^ Department of General Surgery, The Affiliated Hospital of Qingdao University, Qingdao, P.R. China

**Keywords:** interleukin-35, colon cancer, β-catenin, IL-35

## Abstract

Colon cancer development is closely related to inflammation. Thus, we conducted the present investigation to study the effects of IL-35 (Interleukin 35), a newly identified anti-inflammatory factor, on colon cancer development. We first evaluated the IL-35 expression in 186 pairs of colon cancer samples and paired adjacent normal tissues by qPCR, ELISA (Enzyme-linked immunoassay) and tissue microarrays. Then the role of IL-35 on patient survival rates, colon cancer progression and their sensitivity to chemotherapy drugs were assessed. IL-35 was barely expressed in the colon cancer tissue but highly expressed in the adjacent normal tissue. The down-regulation of IL-35 was significantly associated with the AJCC (American Joint Committee on Cancer) stage, nodal involvement, invasion depth, distant metastasis, differentiation and it was also shown to be an independent prognostic indicator of both disease-free and overall survivals for colon cancer patients. Overexpression of IL-35 in colon cancer cell suppressed cell migration, invasion, proliferation, colony formation and cancer stem cells through suppressing β-catenin. IL-35 inhibited colon tumor formation in the mice model and sensitize the cancer cell to chemotherapy drugs. Our results showed that IL-35 shows an inhibitory effect in colon cancer development and is a novel prognostic indicator. Therefore, IL-35 might be a potential therapeutic target.

## INTRODUCTION

Colon cancer is one of the most prevalent malignancies worldwide [1]. It is estimated that over half of the patients with colon cancer had developed distant metastasis [2]. Major progress has been made in cancer detection and treatment during the past decades; however, the 5-year survival rate for colon cancer patients is still low [1, 2]. Thus, identifying more biomarkers, uncovering the mechanisms underlying colon cancer development and novel therapeutic targets development are necessary.

Colon cancer development is a multistage process, which originated from normal mucosa, and then adenomatous polyps, carcinoma, and finally invasive and metastatic carcinoma [3]. Previous studies have demonstrated that multiple mechanisms are responsible for the colon cancer development. Recently, growing evidences indicate that immune mechanisms are crucial in colon cancer progression [4]. Dysregulated inflammatory response and pro-inflammatory cytokines are related to tumor development, including growth, metastasis, apoptosis and angiogenesis [5]. Interleukin-35 (IL-35) is a newly discovered cytokine with anti-inflammatory and immune inhibitory effects [6]. IL-35 consists of two subunits, EBI3 (Epstein-Barr virus induced gene 3) and p35 [6].

Recently, it has been shown that IL-35 plays an important role in tumor progression, including lung cancer [7], pancreatic cancer [8], nasopharyngeal carcinoma [9], gastric cancer [10], hepatocellular carcinoma [11–13], breast cancer [14, 15], renal cell carcinoma [16] and colorectal cancer [17, 18]. However, the underlying mechanisms of IL-35 in colon cancer remain unknown. Thus, we conducted the present investigation to study the role of IL-35 in colon cancer. The results here showed that IL-35 might have inhibitory effects on colon cancer development and also is a novel prognostic indicator. Therefore, IL-35 might be a potential therapeutic target.

## RESULTS

### Decreased IL-35 expression in human colon cancer

**Figure 1:**
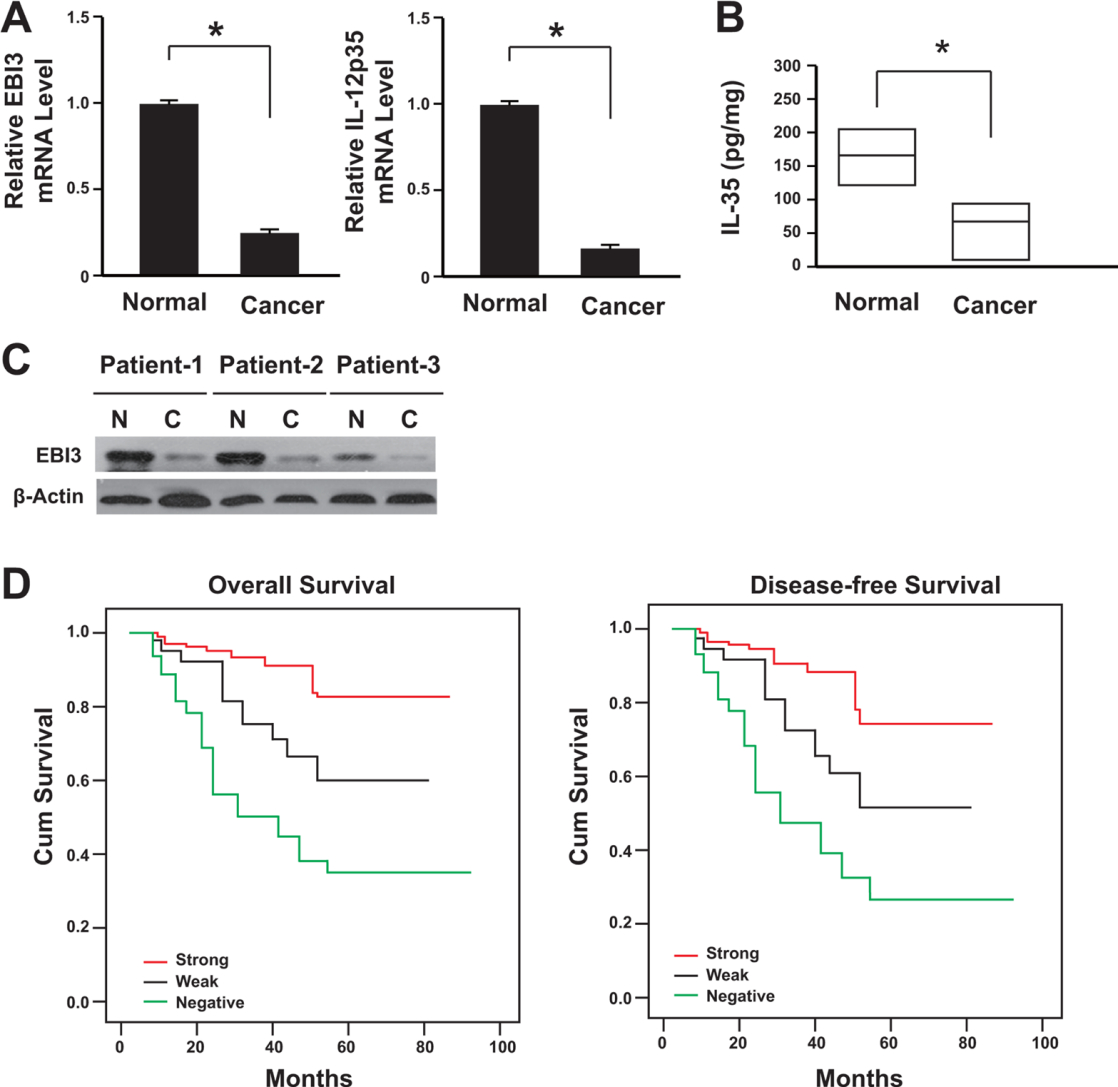
Lack of IL-35 expression correlated with the poor prognosis of colon cancer (**A**) IL-35 mRNA levels in cancer tissues and adjacent normal tissues were determined by real-time PCR (*n* = 186). *P < 0.05. (**B**) IL-35 protein levels in cancer tissues and adjacent normal tissues were determined by ELISA (*n* = 186). Result is depicted as box plots; middle line indicates median; bottom of box, 25th percentile; and top of box, 75th percentile. *P < 0.05. (**C**) Representative figure for IL-35 protein levels in cancer tissues and adjacent normal tissues were determined by western blot. N: adjacent normal tissues; C: cancer tissues. (**D**) Kaplan-Meier survival curve of patients with negative, weak or strong expression of IL-35.

A total of 186 cases with colon cancer were followed. All these patients had received no pre-operation chemotherapy. They were given the same radical operation and underwent the same adjuvant chemotherapy after the surgery. The IL-35 expression was firstly analyzed in 186 colon specimens. The data showed that the IL-35 expression decreased at both mRNA and protein levels in colon cancer tissues comparing with its paired adjacent non-cancerous tissues (Figure 1A and 1B), and this was further confirmed by western blot analysis (Figure 1C).

Tissue microarray and immunohistochemical analysis showed that the lack of IL-35 expression was correlated with lymph nodes metastasis significantly (Table 1). The lack of IL-35 expression also showed a positive association with the AJCC stage (*P* = 0.011), differentiation (*P* = 0.001), nodal involvement (*P* = 0.009), invasion depth (*P* = 0.018) and distant metastasis (*P* = 0.002). IL-35 receptor is comprised of IL-12Rβ2 (Interleukin 12 receptor, beta 2 subunit) and IL-27Rα (Interleukin 27 receptor, alpha subunit) [19]. We also analyzed these two subunits of IL-35 receptor and data showed that there was no significant difference between tumor and paired normal tissues (Supplementary Figure 1). Furthermore, the β-catenin expression was significantly elevated in the tumor cells (Supplementary Figure 2).

**Table 1:** Association between clinicopathological features and IL-35 protein expression

	*N*	IL-35 expression			
		Strong (*n* = 62, %)	Weak (*n* = 54, %)	Negative (*n* = 70, %)	*P* value
Age, years					0.393
< 65	72	34.0	37.0	45.0	
≥ 65	114	66.0	63.0	55.0	
Gender					0.521
Male	80	48.4	40.7	39.1	
Female	106	51.6	59.3	60.9	
Location					0.651
Right	74	41.9	35.2	42.0	
Transverse	18	11.2	11.1	7.2	
Left	19	4.8	13.0	13.1	
Sigmoid colon	75	42.1	40.7	37.7	
T stage					0.018*
T1	8	3.2	5.5	3.0	
T2	21	16.1	13.0	5.7	
T3	72	46.9	42.5	29.1	
T4	85	33.8	39.0	62.2	
N stage					0.009*
N0	96	67.7	48.1	40.7	
N1	58	25.7	33.3	34.7	
N2	32	6.6	18.6	24.6	
M stage					0.002*
M0	168	96.8	96.3	81.3	
M1	18	3.2	3.7	18.7	
AJCC stage					0.011*
I	22	17.7	11.0	7.2	
II	71	46.8	37.0	31.9	
III	75	32.2	48.0	42.0	
IV	18	3.3	4.0	18.9	
Differentiation					0.001*
High	90	66.0	50.0	32.0	
Moderate	68	27.4	36.0	41.0	
Low	28	6.6	14.0	27.0	
Vascular invasion					0.422
Yes	173	96.8	92.6	91.4	
No	13	3.2	7.4	8.6	

*P < 0.05 indicates a significant association among the variables.

Patient follow-up was performed for all patients. And during the follow-up, 62 patients had died and 75 experienced recurrence. Kaplan-Meier curves with a log-rank test for overall survival (OS) and disease-free survival (DFS) was conducted to assess the predictive role of IL-35 for distant metastasis.

After the therapy, group with IL-35 negative patients showed a significant difference comparing with IL-35 positive group (Figure 1D). Patients with IL-35 negative tumors subsequently developed more metastasis or recurrence than the patients with IL-35 positive tumors (*P* < 0.01). When compared with patients with IL-35 positive tumors, the patients with IL-35 negative tumors showed significantly lower DFS and OS rates (Figure 1D, *P* < 0.001).

Univariate analysis showed that patients with IL-35 negative colon tumors showed a significantly lower DFS and OS than the patients with IL-35 positive tumors (Table 2). Furthermore, the lack of IL-35 expression was an independent marker for predicting cancer recurrence (Table 3).

**Table 2:** Univariate Cox proportional hazards model for disease-free survival (DFS) and overall survival (OS)

	DFS			OS		
	HR	95% CI	*P* value	HR	95% CI	*P* value
Age, years						
< 65	—			—		
≥ 65	1.009	0.659–1.841	0.714	0.934	0.530–1.644	0.812
Gender						
Male	—			—		
Female	1.013	0.617–1.662	0.961	1.546	0.860–2.776	0.145
Tumor location						
Right	—			—		
Transverse	0.816	0.309–2.155	0.681	0.789	0.267–2.334	0.669
Left	1.392	0.619–3.130	0.423	1.291	0.512–3.252	0.589
Sigmoid colon	1.251	0.718–2.176	0.431	1.145	0.614–2.135	0.671
T stage						
T1	0.494	0.119–2.049	0.331	0.988	0.299–3.259	0.984
T2	0.203	0.063–0.660	0.006*	0.394	0.138–1.130	0.083
T3	0.484	0.284–0.827	0.004*	0.521	0.280–0.966	0.019*
T4	—			—		
N stage						
N0	—			—		
N1	5.887	3.025–11.456	< 0.001*	4.157	2.009–8.602	< 0.001*
N2	15.914	7.781–32.545	< 0.001*	13.193	6.149–28.307	< 0.001*
AJCC stage						
I	—			—		
II	1.108	0.305–4.027	0.877	0.667	0.201–2.215	0.508
III	6.823	2.097–22.199	0.001*	3.401	1.184–9.771	0.023*
IV	49.185	12.615–191.764	< 0.001*	40.074	11.257–142.668	< 0.001*
Differentiation						
High	—			—		
Moderate	1.315	0.750–2.306	0.341	1.458	0.764–2.780	0.253
Low	3.577	1.885–6.786	< 0.001*	4.358	2.140–8.872	<0.001*
Vascular invasion						
Yes	4.901	2.469–9.721	< 0.001*	4.638	2.152–9.997	<0.001*
No	—			—		
IL-35 expression						
Positive	—			—		
Weak	2.598	1.194–5.653	0.011*	2.117	0.862–5.196	0.102
Negative	6.118	3.004–12.462	< 0.001*	6.348	2.875–14.014	<0.001*

*P < 0.05 indicates a significant association among the variables.

**Table 3:** Multivariate Cox proportional hazards model for DFS and OS

	DFS			OS		
	HR	95 % CI	*P* value	HR	95 % CI	*P* value
IL-35 expression	2.796	1.919–4.161	< 0.001*	2.659	1.711–4.223	< 0.001*
T stage	1.701	1.129–2.541	0.008*	3.981	1.854–9.173	< 0.001*
N stage	3.698	2.049–6.701	< 0.001*	3.321	1.813–6.203	< 0.001*
M stage	4.402	1.299–14.551	0.011*	8.001	2.403–26.815	< 0.001*

*P < 0.05 indicates a significant association among the variables.

Taken together, these results indicated that the low expression of IL-35 might be associated with colon cancer progression and poor clinical outcomes.

### The anti-tumor effects of IL-35 on colon cancer cell lines are in a dose-dependent manner

**Figure 2:**
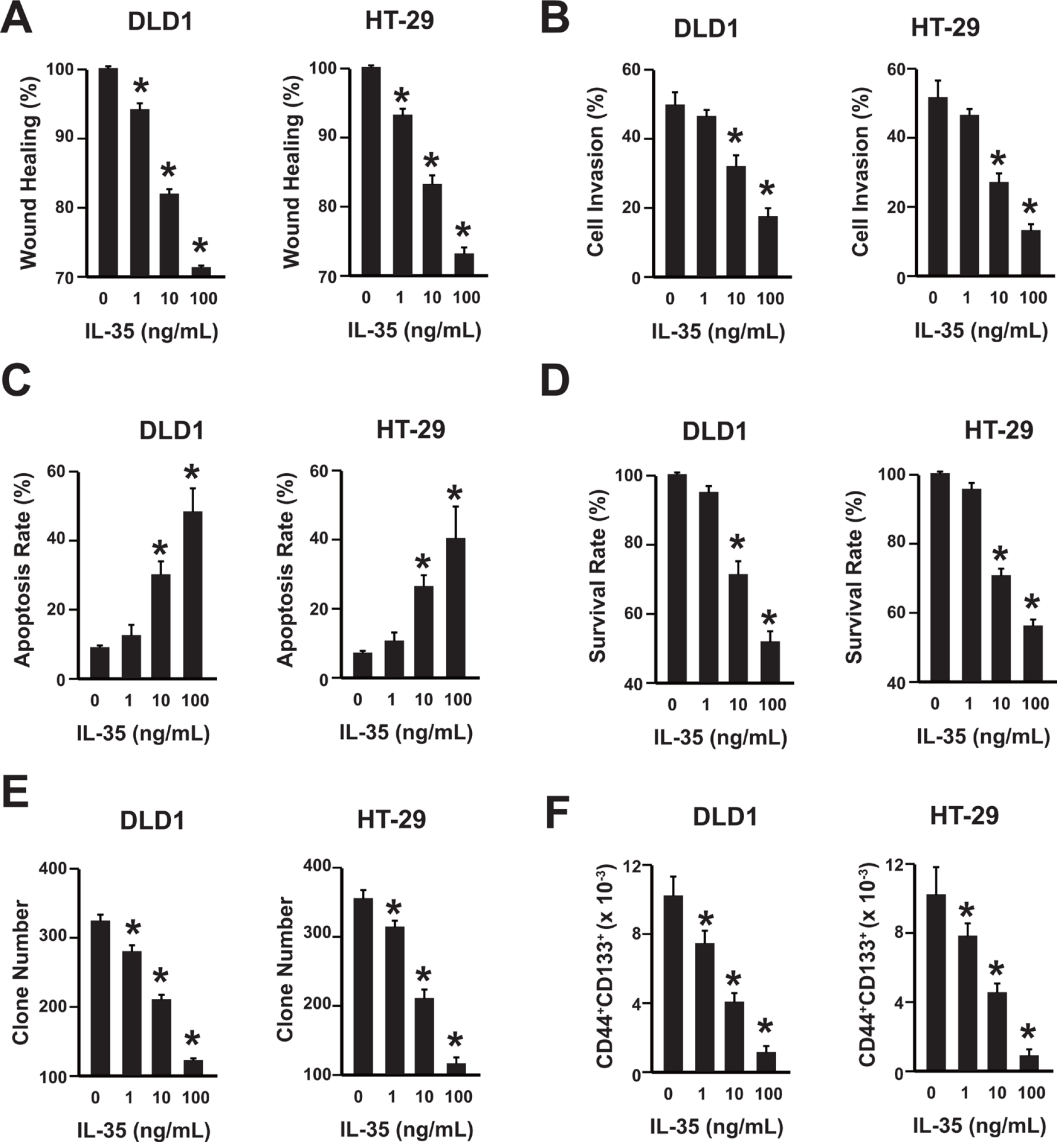
IL-35 suppresses colon cancer in a dose-dependent manner (**A**) Wound healing assay of DLD1 and HT-29 cells with different concentrations of rhIL-35 protein (0, 1, 10, 100 ng/mL). *n* = 3. *P < 0.05. (**B**) Cell invasion assay of DLD1 and HT-29 cells with different concentrations of rhIL-35 protein (0, 1, 10, 100 ng/mL). *n* = 3. *P < 0.05. (**C**) Analysis of colon cancer cell apoptosis following treatment of rhIL-35. DLD1 and HT-29 cells were treated at the indicated doses, harvested, and stained with Annexin V-FITC and 7-AAD. Annexin V-FITC-positive apoptotic cells were determined by flow cytometry. *n* = 3. *P < 0.05. (**D**) The survival rate of DLD1 and HT-29 cells treated with different concentrations of rhIL-35 (0, 1, 10, 100 ng/mL) were analyzed. *n* = 3. *P < 0.05. (**E**) The clone formation number of DLD1 and HT-29 cells treated with different concentrations of rhIL-35 (0, 1, 10, 100 ng/mL) were analyzed. *n* = 3. *P < 0.05. (**F**) The percentage of CD44^+^CD133^+^ cancer stem cells of DLD1 and HT-29 cells treated with different concentrations of rhIL-35 (0, 1, 10, 100 ng/mL) were analyzed. *n* = 3. *P < 0.05.

To further uncover the role of IL-35 in colon cancer development, cell migration, invasion, apoptosis, proliferation and cancer stem cell were analyzed in two human colon cancer cell lines, DLD1 and HT-29, after the rhIL-35 (recombinant human IL-35) treatment. IL-35 inhibited the migration and invasion of DLD1 and HT-29 cells in a dose-dependent manner (Figure 2A, 2B and Supplementary Figure 3). Additionally, IL-35 increased the apoptosis of DLD1 and HT-29 cells in a dose-dependent manner (Figure 2C). Moreover, IL-35 decreased the proliferation of DLD1 and HT-29 cells (Figure 2D). Furthermore, their clone formation capability and the percentage of colon cancer stem cell (CD44^+^CD133^+^ population) within colon cancer cells were also reduced by IL-35 (Figure 2E and 2F, Supplementary Figure 3) [20–22]. Stemness factors analysis showed that IL-35 could suppress their expression significantly (Supplementary Figure 4).

Therefore, IL-35 could suppress the migration, invasion and proliferation, and promote the apoptosis of colon cancer cells. Furthermore, the IL-35 could reduce the number of cancer stem cells.

### IL-35 inhibits β-catenin expression in colon cancer cells

**Figure 3:**
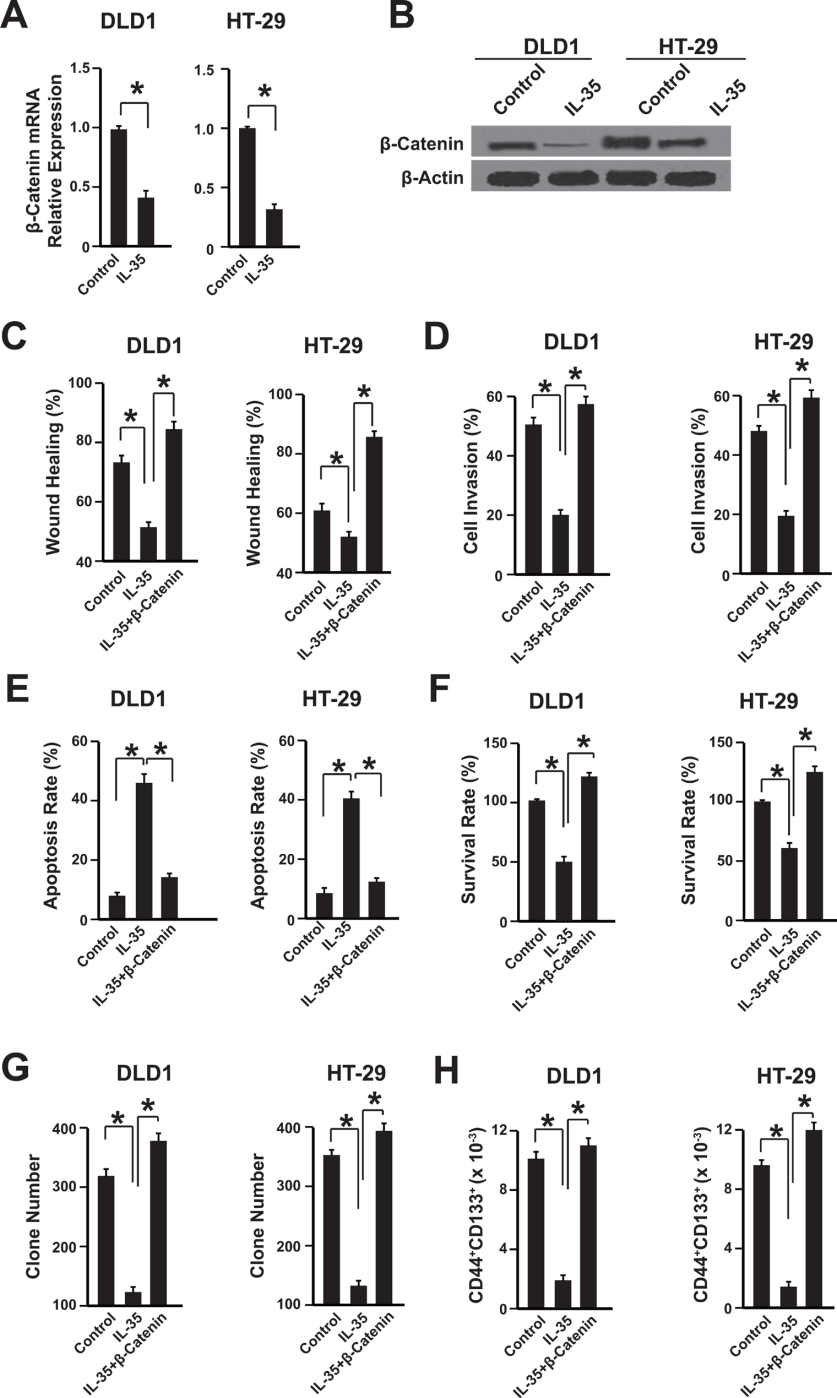
IL-35 inhibits β-catenin expression in colon cancer cells (**A**) The mRNA level of β-catenin was measured by qPCR. DLD1 and HT-29 cells were treated with 100 ng/mL rhIL-35. *n* = 3. *P < 0.05. (**B**) The protein level of β-catenin was measured by western blot. DLD1 and HT-29 cells were treated with 100 ng/mL rhIL-35. *n* = 3. *P < 0.05. (**C**–**H**) The wound healing, cell invasion, apoptosis, survival rate, clone formation capability and the percentage of cancer stem cells of cells overexpressing β-catenin or empty vector were analyzed. Cells were treated with 100 ng/mL rhIL-35. *n* = 3. * *P* < 0.05.

We further investigated the underlying mechanism of anti-tumor effects of IL-35 in colon cancer cells. The expressions of β-catenin in DLD1 and HT-29 cells were analyzed after rhIL-35 treatment, because of the important role of β-catenin in colon cancer development [23]. The mRNA and protein levels of β-catenin were suppressed by IL-35 in these two cell lines (Figure 3A and 3B). To further confirm the correlation between IL-35 andβ-catenin signaling pathway, we transfected the DLD1 and HT-29 cells with plasmids overexpressing IL-35, β-catenin or them together. Compared with the control group, the effects of IL-35 on cell migration, invasion, apoptosis, proliferation and cancer stem cells were abolished in β-catenin overexpressing cells (Figure 3C–3H). Taken together, these results suggest that IL-35 may inhibit colon cancer cells via β-catenin pathway.

### Overexpression of IL-35 suppresses colon cancer development *in vivo* and increases the sensitivity to chemotherapeutic drugs

**Figure 4:**
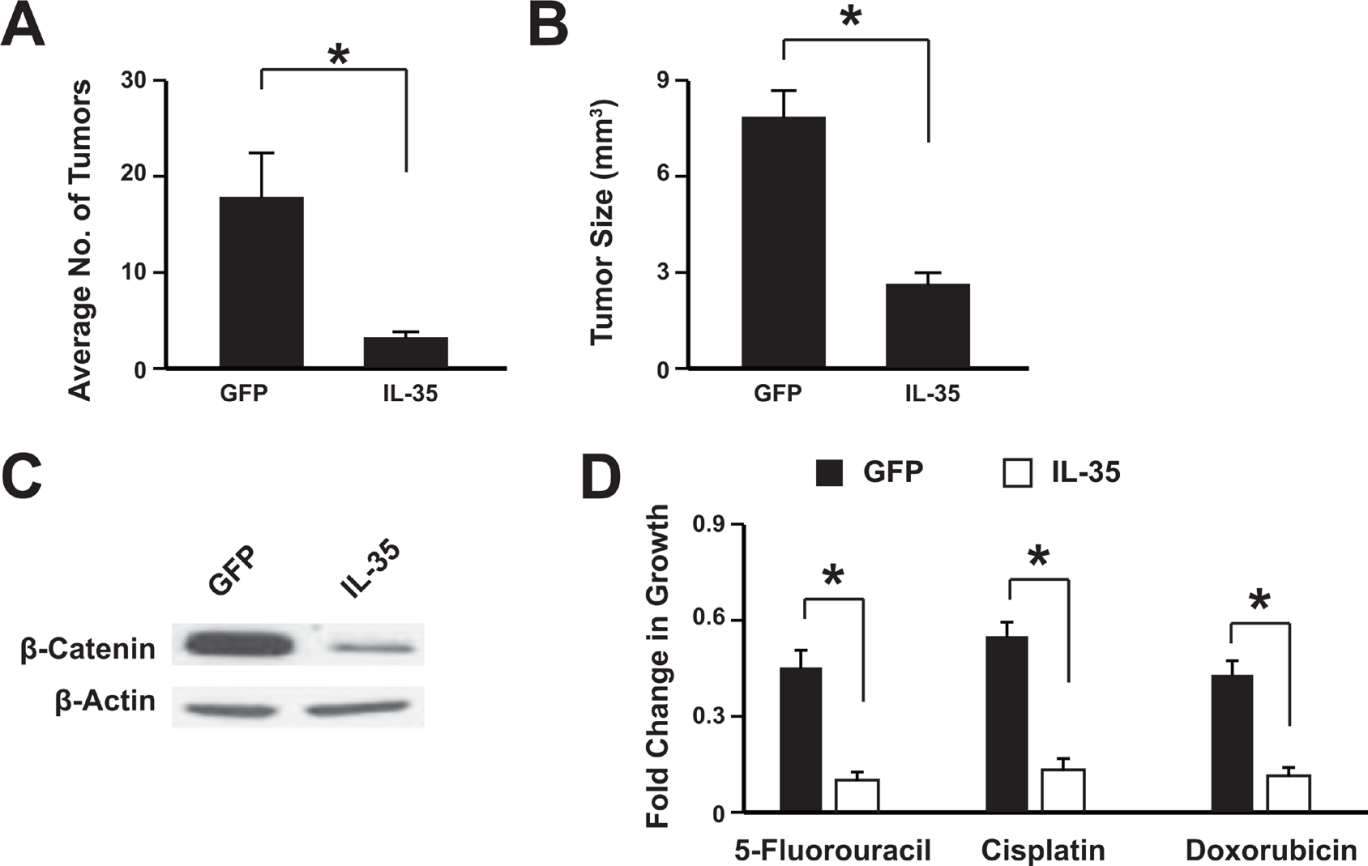
IL-35 suppresses colon tumorigenesis *in vivo* The adeno-associated virus expressing IL-35 or GFP was administrated via tail-vein injection. The average tumor number per mice (**A**) and the tumor size (**B**) were analyzed. Western blot showed that IL-35 suppressed β-catenin expression in the mice tumor (**C**). Tumor cells were isolated from the mice and then subjected to chemotherapeutic drugs treatment (**D**). *n* = 12. *P < 0.05.

To investigate the role of IL-35 in tumorigenesis, the colonic tumors were induced in mice with azoxymethane and DSS treatment. Then the adeno-associated virus expressing IL-35 or GFP was administrated via tail-vein injection. The results showed that mice treated with IL-35 had significantly less and smaller tumors than control mice (Figure 4A and 4B). The overexpression of IL-35 was validated with western blot (Supplementary Figure 5) and it could suppress the β-catenin expression (Figure 4C). To further confirm the anti-tumor effect of IL-35, the IL-35 was over-expressed in the HT29 cells and then implanted in the mice. Data showed that IL-35 could significantly inhibit the tumor formation (Supplementary Figure 6). Tumor cells were isolated from the mice and then subjected to chemotherapeutic drugs. Data showed that IL-35 treatment sensitize the colon cancer to these drugs, including 5-Fluorouracil, Cisplatin and Doxorubicin (Figure 4D). These results showed that IL-35 could suppress tumor development *in vivo* and might sensitize the tumors to chemotherapy.

## DISCUSSION

Despite the current treatments for colon cancer improve the survival significantly; the development of drug resistance still occurs in a great number of patients determining recurrence. Early diagnosis of colon cancer can improve survival rate, however, most patients are diagnosed at an advanced stage [1, 2]. Therefore, more efforts should be made to uncover the underlying mechanisms and develop novel therapeutic targets.

IL-35 has been classified as an anti-inflammatory factor and immune suppressor [6]. Recent studies demonstrated that IL-35 plays an important role in cancer development. However, how IL-35 affects the colon cancer development remains unknown. The function of IL-35 in tumor is largely unknown. Inflammation is the seventh hallmark of tumors [24], inferring that IL-35 might influence inflammation related tumors. Here, we provided the evidences that IL-35 suppresses the colon cancer progression through β-catenin suppression.

We first studied the expression pattern of IL-35 protein in colon cancer patients. It showed that IL-35 expression was decreased in cancer tissues comparing with paired normal tissues. Further analysis showed that the lack of IL-35 tightly associated with cancer metastasis and poor survival, indicating that IL-35 could be developed as novel prognostic marker to predict tumor recurrence.

To further validate the clinical study, we treated colon cancer cell line DLD1 and HT-29 with different concentration of IL-35. We found that IL-35 suppressed migration, invasion and cell growth of colon cancer *in vitro*. In the meantime, IL-35 also promoted colon apoptosis and reduced cancer stem cells. Aberrant β-catenin activation contributes to colon cancer progression [23], thus we then assessed the β-catenin expression. We found that IL-35 down-regulated the β-catenin expression at both mRNA and protein level. In our study, the reduced expression of β-catenin might contribute to the antitumor effects of IL-35. Furthermore, we also confirmed the anti-tumor effects of IL-35 in the mice model of colon cancer and demonstrated that IL-35 could sensitize the colon cancer to chemotherapeutic drugs.

In conclusion, our data showed that IL-35 expression is lower in the colon cancer. By inhibiting β-catenin expression, IL-35 impeded the colon cancer development. Therefore, IL-35 might be a potential therapeutic target in colon cancer. However, the underlying mechanism of IL-35 reducing β-catenin expression in colon cancer is not clear. The reason for the divergent results between our study and previous reports remains unclear [17, 18], which also occurred to hepatocellular carcinoma [11–13]. Our results here are in accordance with previous report that low expression of IL-35 might contribute to the cancer progression [12]. And further studies are necessary to uncover the anti-tumor mechanisms of IL-35 in colon cancer.

## MATERIALS AND METHODS

### Patients

186 colon cancer patients were recruited. They had primary colon cancer and did not receive any preoperative therapy at the Department of General Surgery of The Affiliated Hospital of Qingdao University between January 2005 and December 2009. Matched pairs of specimen were collected immediately after tumor removal. Cancer and paired normal tissues had the distance of more than 5 cm. Specimens have been divided into two aliquots: half were frozen immediately for RNA and protein extraction; half were fixed with formalin for TMA ((Tissue Microarrays)) construction. The study was approved by the Ethics Committee of The Affiliated Hospital of Qingdao University (QDUH 2005023821, Jan. 2005). Written informed consent was obtained from all patients.

### RNA extraction and quantitative polymerase chain reaction (qPCR)

RNA was extracted with Trizol (Invitrogen, Carlsbad, CA, USA) and cDNA was prepared with iScript™ cDNA Synthesis kit (Bio-Rad, USA). PCR primers used for qPCR were as follows: for EBI3, sense: 5′- GCTTCGTGCCTTTCATAACAG-3′ and anti-sense: 5′- GCTCCCACTGCACCTGTA −3′; IL-12p35, sense: 5′- ACATGCTGGCAGTTATTGATGA −3′ and anti-sense: 5′- TGAAGAAGTATGCAGAGCTTGAT −3′;β-actin, sense: 5′-CCTGACTGACTACCTCATGAAG-3′ and anti-sense: 5′-GACGTAGCACAGCTTCTCCTTA-3′. qPCR amplification reaction was prepared with the SYBR Green PCR kit (Bio-rad, USA) and 7500 fast Real-Time PCR system (Applied Biosystems, USA). mRNA levels were calculated by the 2^−ΔΔct^ method.

### Western blotting

Tissues and cultured cells were lysed in RIPA buffer with protease inhibitors. The protein was quantified, separated by 10% SDS-PAGE, transferred onto PVDF membranes, and then incubated with primary antibodies against EBI3 (PA5-23297, Thermo Scientific, USA), IL-12p35 (MAB1570, R&D Systems), β-catenin (ab32572, Abcam, Cambridge, UK) and β-actin (ab8227 Abcam, Cambridge, UK). The membrane was extensively washed and then incubated with secondary antibody, visualized by an Alpha Imager (Alpha Innotech, San Leandro, CA).

### Enzyme-linked immunoassay (ELISA)

IL-35 was detected in the tumor homogenate and culture supernatants with human IL-35 ELISA kit (Abcam, Cambridge, UK) according to the manufacturer's instructions.

### TMA (Tissue Microarrays) construction and immunohistochemistry

Formalin-fixed, paraffin-embedded samples, including cancer tissue and paired normal tissue, were analyzed. Three cores from each tissue were arrayed. TMAs were created with Tissue Microarrayer (Beecher Instruments, Sun Prairie, WI, USA). All samples were examined by three pathologists.

For IL-35 immunostaining, the antigen retrieval and endogenous peroxidase inhibition were performed. The sections were then blocked with fetal calf serum, incubated with mouse anti-human IL-35 antibody (6H15L18, Thermo Scientific, USA), IL12RB2 antibody (ab67365, Abcam), IL-27Rα Antibody (J126, Santa Cruz Biotechnology) and then secondary antibody. They were visualized with 3, 3-diaminobenzidine (DAB) and Mayer's hematoxylin staining.

The intensity score was evaluated by three independent observers blindedly. The intensity score was defined as 0, 1, 2 and 3 for negative, weak, moderate and strong staining, individually. The extent score was defined as 0, 1, 2 and 3 for negative, less than 10% positive, 10%–50% positive and more than 50% positive, individually. Then the intensity and extent score were summed up. The total score of 0 to 2 was defined as negative expression, 3 to 4 as weak expression and 5–9 as strong expression.

### Recombinant human IL-35 protein expression

Interleukin-35 gene (homo species, EBI3 and IL-12p35 individually) was amplified with high fidelity PCR and ligated into the expression vector. The protein was expressed in a stable prokaryotic expression system. The protein was purified with His Trap HP (GE) and verified via immunoblot analysis with anti-human EBI3 and IL-12p35 antibodies.

### Cell culture

The DLD1 and HT-29 human colon cell lines were purchased from the American Type Culture Collection (ATCC, USA) and cultured in DMEM (GIBCO) plus 10 % FBS. Recombinant human IL-35 (rhIL-35, EBI3: IL-12p35 = 1:1) was used to treat DLD1 and HT-29 cells for 24 hrs.

### Cell viability assay

Cell viability was assessed with CCK-8 method (Beyotime, China). Briefly, cells were treated with CCK-8 solution for 1 hour, and then the OD_450_ values were read with a microplate reader (Bio-Tek Company, USA). The experiment was repeated for three times.

### Cell apoptosis assay

Cell apoptosis assay was conducted by flow cytometry with the Annexin V-FITC Apoptosis Detection Kit (Roche, China). Briefly, cells were trypsinized, neutralized and washed with PBS. Cells were resuspended in binding buffer and incubated with Annexin V-FITC and propidium iodide for 10 min. Then cells analyzed by BD FACSCalibur (BD Bioscience, USA) and analyzed by Flowjo software (FlowJo, USA). The experiment was repeated three times.

### Cell migration assay

Cell migration ability was assessed by wound-healing assay. Briefly, a wound line was created with the pipette tip when cells reach 80–90% confluence. The wound area was recorded and also 24 hrs later. Cell migration = area of cell migrated comparing to the area of the initial wound. All experiments were repeated three times.

### Transwell invasion assay

Invasive ability of cells was determined within a transwell system. 10 × 10^4^ cells were seeded onto the Matrigel coated transwell membrane and cultured for 48h. Then the cells migrated to the lower surface was counted under a microscope. Both cells migrated and not migrated were counted for normalization.

### Clonogenic assays

The cells were plated at 1 × 10^3^ cells per well in 6-well plates with agarose. 10 days later, the plates were fixed and stained with 0.2% crystal violet (w:v) in 10% buffered formalin. Colony number was counted.

### Flow Cytometry

Cells were harvested and resuspended in PBS containing 0.1% BSA. The cells were incubated at 4°C for 30 minutes with anti-CD44-APC (559942, BD Biosciences) and anti-CD133-PE (130-101-652, MiltenyiBiotec) antibodies, or isotype controls. The cells were washed three times and then resuspended in PBS containing 0.1% BSA plus 2 μg/mL propidium iodide (PI). Flow cytometry analysis was conducted with C6 FACS (BD Biosciences).

### Animal study

6–8 weeks old C57 mice were housed in specific pathogen-free conditions. For induction of colonic tumors, mice were first administered an intraperitoneal injection of azoxymethane (7.4mg/kg, Sigma-Aldrich, USA); one week later, 1% DSS was first administered for 7 days in drinking water, then followed by drinking distilled water for 3 weeks. Finally the mice were killed. All polypoid or flat elevated lesions that developed were histo-pathologically counted by observation of a longitudinal paraffin section with H&E staining. Tumor size was measured with fine digital calipers and calculated as width^2^ × length/2. The study was approved by the Research Ethics Committee of The Affiliated Hospital of Qingdao University (QDUA 2015365214, Aug. 2015).

### Production and *in vivo* delivery of adeno-associated virus

Vector construction, production, and *in vivo* delivery of adeno-associated virus (AAV) were performed based on the AAV helper-free system (Agilent). The recombinant adenoviral vector pAAV-IL35 (EBI3-2A-IL-12p35) was constructed by cloning the cDNA encoding region into pAAV-ITR. The vector pAAV-GFP encoding green fluorescence protein was used as a negative control. Recombinant AAVs were produced by HEK293 cells (ATCC) transfected with pAAV-ITR vectors together with pAAV-RC and pHelper plasmids, and then purified by discontinuous iodixanol gradient centrifugation. Purified recombinant AAVs were concentrated and desalted by centrifugation through Amicon Ultra 30K filters (Millipore). For *in vivo* delivery, recombinant AAVs equivalent to 1.0 × 10^12^ viral genome copies were delivered though mouse tail vein.

### Chemotherapy assay

Cells were treated with 5-Fluorouracil (1.5μM, Sigma), Cisplatin (1.5 μM, Sigma), and Doxorubicin (30 nM, Sigma) for 24 hours. Then the cells were plated at 1 × 10^3^/well in 6-well plates. 10 day later, cells were fixed and stained with crystal violet. Dye was then solubilized with 1% SDS and OD_590_ was measured.

### Statistical analysis

Data were expressed as mean (±SE) and analyzed by a SPSS software package (SPSS Standard version 13.0, SPSS Inc, USA). Differences between variables were assessed by the Chi-square test. Survival analysis of patients with colorectal cancer was calculated by Kaplan-Meier analysis. A log rank test was used to compare different survival curves. The univariate and multivariate hazard ratios were calculated with Cox proportional hazards model. Unpaired Student's t test and one way ANOVA were used as appropriate to assess the statistical significant of difference. *P* values under 0.05 were considered statistically significant.

## CONCLUSIONS

Our results demonstrated that IL-35 is decreased in human colon cancer and IL-35 is capable of exerting anti-tumor activity by suppressing the β- catenin expression.

## SUPPLEMENTARY MATERIALS FIGURES AND TABLES



## References

[1] Siegel RL, Miller KD, Jemal A (2016). Cancer statistics, 2016. CA Cancer J Clin.

[2] Siegel R, Desantis C, Jemal A (2014). Colorectal cancer statistics, 2014. CA Cancer J Clin.

[3] Worthley DL, Whitehall VL, Spring KJ, Leggett BA (2007). Colorectal carcinogenesis road maps to cancer. World J Gastroenterol.

[4] Pancione M, Giordano G, Remo A, Febbraro A, Sabatino L, Manfrin E, Ceccarelli M, Colantuoni V (2014). Immune escape mechanisms in colorectal cancer pathogenesis and liver metastasis. J Immunol Res.

[5] Zhong Z, Sanchez-Lopez E, Karin M (2016). Autophagy Inflammation, and Immunity: A Troika Governing Cancer and Its Treatment. Cell.

[6] Collison LW, Workman CJ, Kuo TT, Boyd K, Wang Y, Vignali KM, Cross R, Sehy D, Blumberg RS, Vignali DA (2007). The inhibitory cytokine IL-35 contributes to regulatory T-cell function. Nature.

[7] Nishino R, Takano A, Oshita H, Ishikawa N, Akiyama H, Ito H, Nakayama H, Miyagi Y, Tsuchiya E, Kohno N, Nakamura Y, Daigo Y (2011). Identification of Epstein-Barr virus-induced gene 3 as a novel serum and tissue biomarker and a therapeutic target for lung cancer. Clin Cancer Res.

[8] Nicholl MB, Ledgewood CL, Chen X, Bai Q, Qin C, Cook KM, Herrick EJ, Diaz-Arias A, Moore BJ, Fang Y (2014). IL-35 promotes pancreas cancer growth through enhancement of proliferation and inhibition of apoptosis: evidence for a role as an autocrine growth factor. Cytokine.

[9] Zhang Y, Sun H, Wu H, Tan Q, Xiang K (2015). Interleukin 35 is an independent prognostic factor and a therapeutic target for nasopharyngeal carcinoma. Contemp Oncol (Pozn).

[10] Fan YG, Zhai JM, Wang W, Feng B, Yao GL, An YH, Zeng C (2015). IL-35 over-expression is associated with genesis of gastric cancer. Asian Pac J Cancer Prev.

[11] Fu YP, Yi Y, Cai XY, Sun J, Ni XC, He HW, Wang JX, Lu ZF, Huang JL, Cao Y, Zhou J, Fan J, Qiu SJ (2016). Overexpression of interleukin-35 associates with hepatocellular carcinoma aggressiveness and recurrence after curative resection. Br J Cancer.

[12] Long J, Guo H, Cui S, Zhang H, Liu X, Li D, Han Z, Xi L, Kou W, Xu J, Li TS, Ding Y (2016). IL-35 expression in hepatocellular carcinoma cells is associated with tumor progression. Oncotarget.

[13] Qiu X, Wang X, Song Y, Chen L (2016). Plasma Level of Interleukin-35 as an Independent Prognostic Indicator in Hepatocellular Carcinoma. Dig Dis Sci.

[14] Chen G, Liang Y, Guan X, Chen H, Liu Q, Lin B, Chen C, Huang M, Chen J, Wu W, Liang Y, Zhou K, Zeng J (2016). Circulating low IL-23: IL-35 cytokine ratio promotes progression associated with poor prognosisin breast cancer. Am J Transl Res.

[15] Zhao Z, Chen X, Hao S, Jia R, Wang N, Chen S, Li M, Wang C, Mao H (2017). Increased interleukin-35 expression in tumor-infiltrating lymphocytes correlates with poor prognosis in patients with breast cancer. Cytokine.

[16] Jin L, Xu X, Ye B, Pan M, Shi Z, Hu Y (2015). Elevated serum interleukin-35 levels correlate with poor prognosis in patients with clear cell renal cell carcinoma. Int J Clin Exp Med.

[17] Zeng JC, Zhang Z, Li TY, Liang YF, Wang HM, Bao JJ, Zhang JA, Wang WD, Xiang WY, Kong B, Wang ZY, Wu BH, Chen XD (2013). Assessing the role of IL-35 in colorectal cancer progression and prognosis. Int J Clin Exp Pathol.

[18] Ma Y, Chen L, Xie G, Zhou Y, Yue C, Yuan X, Zheng Y, Wang W, Deng L, Shen L (2016). Elevated level of interleukin-35 in colorectal cancer induces conversion of T cells into iTr35 by activating STAT1/STAT3. Oncotarget.

[19] Wang RX, Yu CR, Dambuza IM, Mahdi RM, Dolinska MB, Sergeev YV, Wingfield PT, Kim SH, Egwuagu CE (2014). Interleukin-35 induces regulatory B cells that suppress autoimmune disease. Nat Med.

[20] Haraguchi N, Ohkuma M, Sakashita H, Matsuzaki S, Tanaka F, Mimori K, Kamohara Y, Inoue H, Mori M (2008). CD133+CD44+ population efficiently enriches colon cancer initiating cells. Ann Surg Oncol.

[21] Galizia G, Gemei M, Del Vecchio L, Zamboli A, Di Noto R, Mirabelli P, Salvatore F, Castellano P, Orditura M, De Vita F, Pinto M, Pignatelli C, Lieto E (2012). Combined CD133/CD44 expression as a prognostic indicator of disease-free survival in patients with colorectal cancer. Arch Surg.

[22] Bellizzi A, Sebastian S, Ceglia P, Centonze M, Divella R, Manzillo EF, Azzariti A, Silvestris N, Montemurro S, Caliandro C, De Luca R, Cicero G, Rizzo S (2013). Co-expression of CD133(+)/CD44(+) in human colon cancer and liver metastasis. J Cell Physiol.

[23] Morin PJ, Sparks AB, Korinek V, Barker N, Clevers H, Vogelstein B, Kinzler KW (1997). Activation of beta-catenin-Tcf signaling in colon cancer by mutations in beta-catenin or APC. Science.

[24] Hanahan D, Weinberg RA (2011). Hallmarks of cancer: the next generation. Cell.

