# Supplementary material for: Interleukin-35 expression is associated with colon cancer progression
Source: Oncotarget. 2017 May 10;8(42):71563–73. doi: 10.18632/oncotarget.17751 (PMC5641072; doi:10.18632/oncotarget.17751)
Supplement: Supplementary file 1 [file oncotarget-08-71563-s001.pdf]

Interleukin-35 expression is associated with colon cancer progression

Supplementary Materials

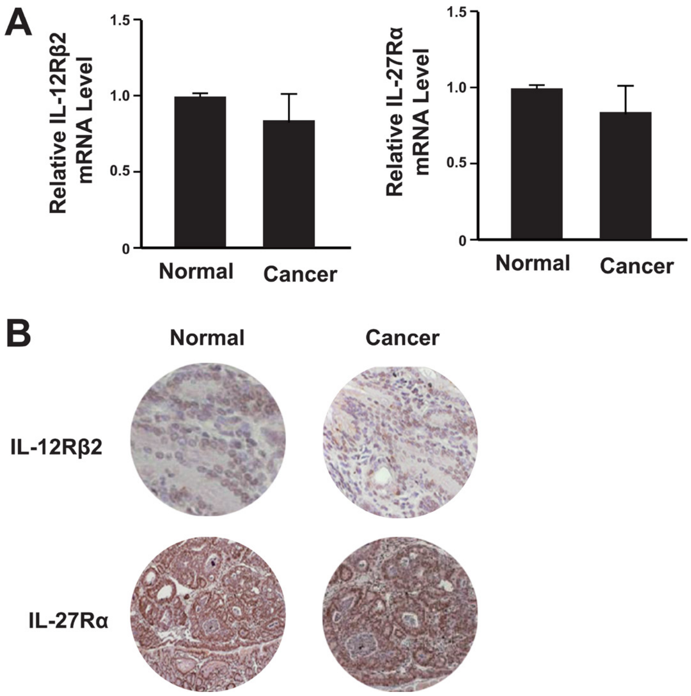

Supplementary Figure 1: IL-35 receptor expression in human colon cancer biopsies and paired normal tissues. (**A**) mRNA level of IL-35 receptor subunits (n = 186). (**B**) Representative color photomicrographs of IL-35 receptor subunits expression.

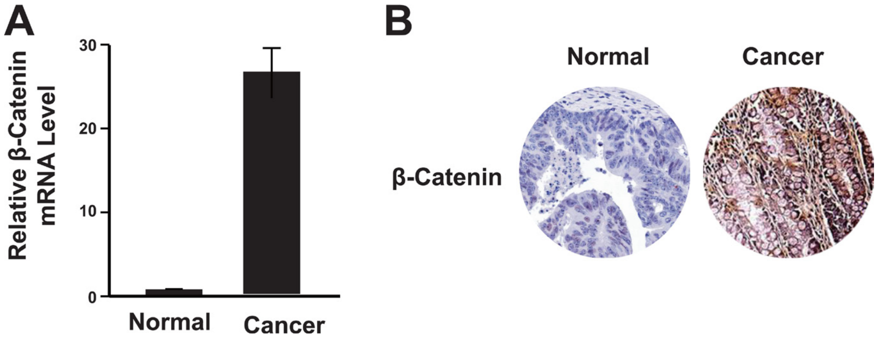

Supplementary Figure 2: The expression level of β-catenin in human colon cancer biopsies and paired normal tissues. (**A**) mRNA level of β-catenin (n = 186). (**B**) Representative color photomicrographs of β-catenin expression.

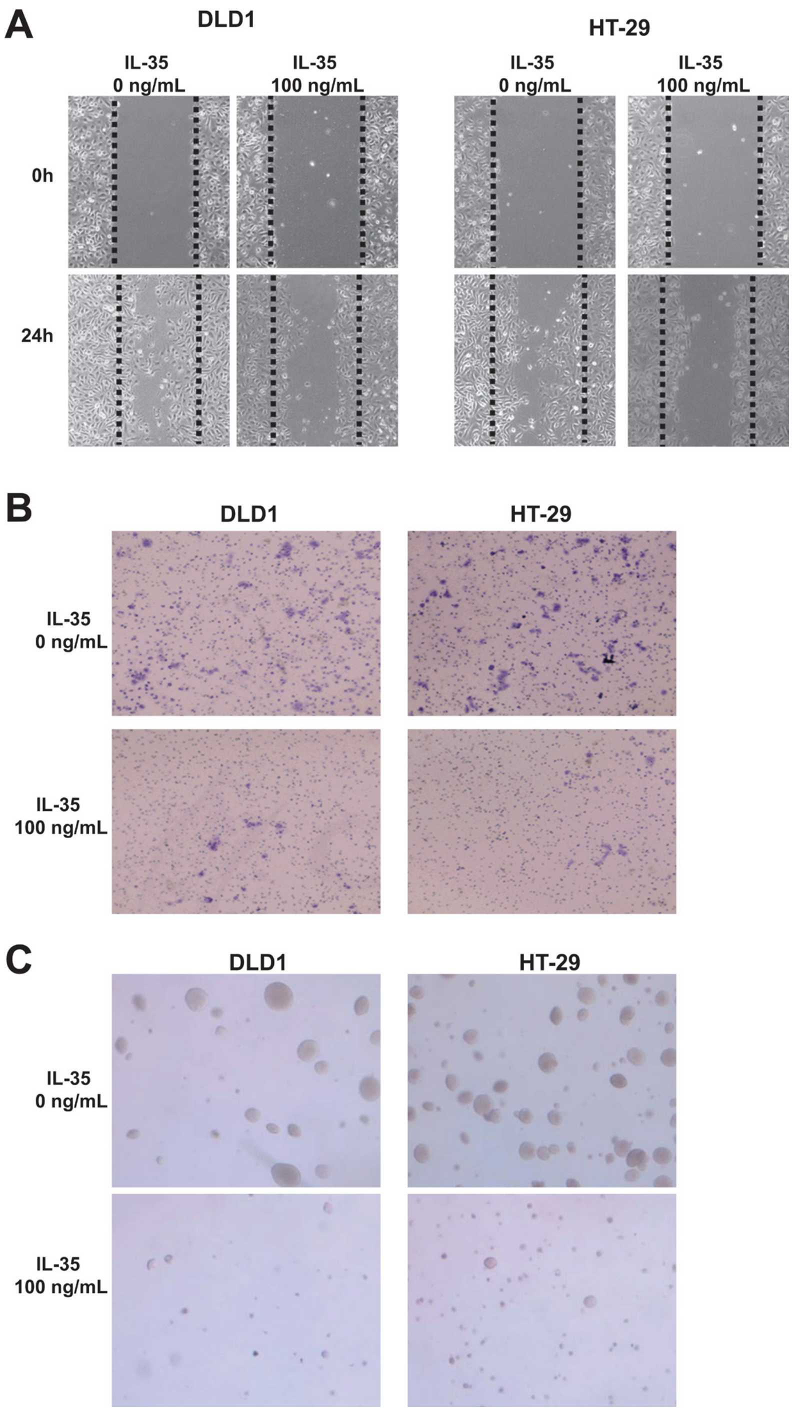

Supplementary Figure 3: The cell migration (**A**), invasion (**B**) and clonogenic formation (**C**) of DLD1 and HT-29 cells with or without IL-35 treatment.

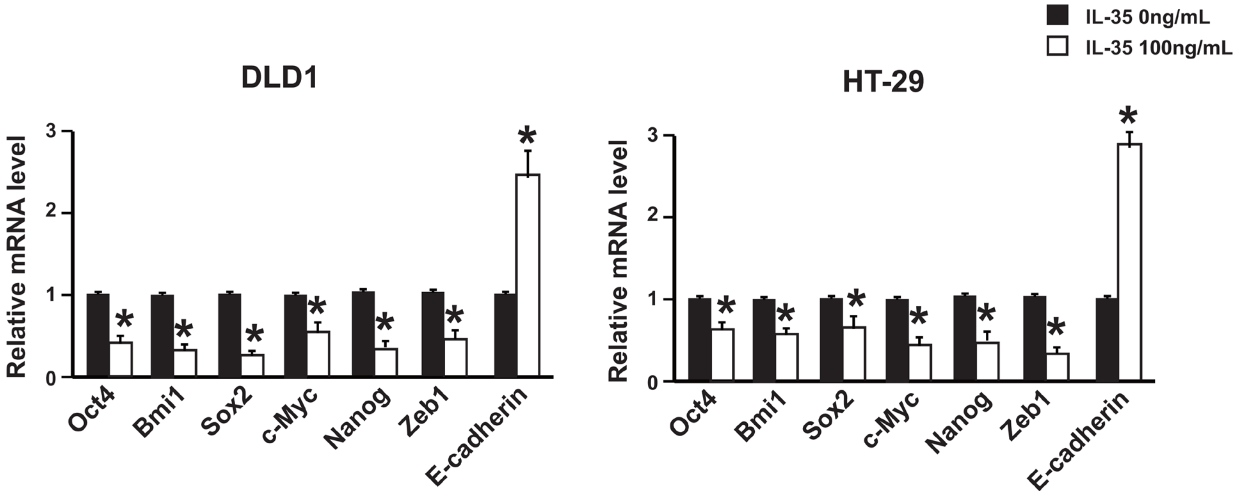

Supplementary Figure 4: The mRNA level of stemness factors in DLD1 and HT-29 cells with or without IL-35 treatment were analyzed by qPCR (*n* = 3).

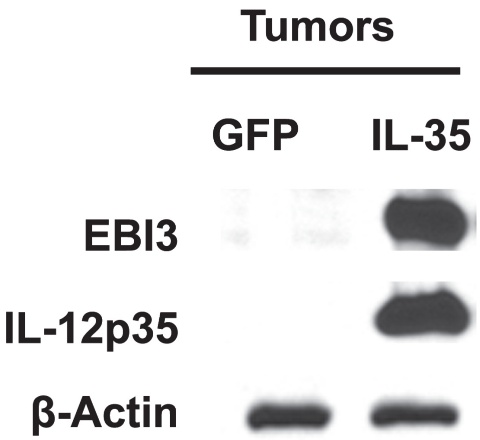

Supplementary Figure 5: Western blot analysis of IL-35 overexpression in mice colon tumors.

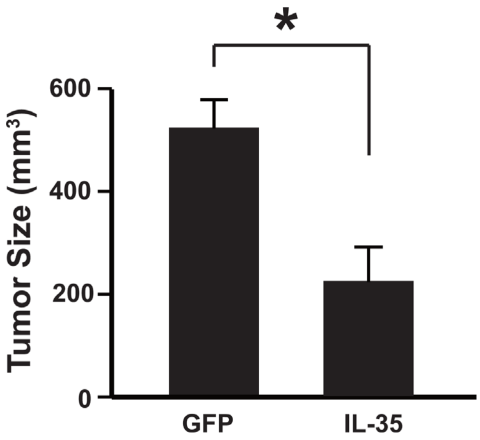

Supplementary Figure 6: Tumor size analysis of the mice transplanted with HT-29 cells overexpressing GFP or IL-35.
